# A bacterial-plant auxin alliance enhances phosphorus acquisition

**DOI:** 10.1093/plphys/kiaf061

**Published:** 2025-02-18

**Authors:** Haiyan Yu, Jiaqi Sun

**Affiliations:** State Key Laboratory of Microbial Technology, Microbial Technology Institute, Shandong University, Qingdao, Shandong 266237, China; Assistant Features Editor, Plant Physiology, American Society of Plant Biologists; The Key Laboratory of Plant Development and Environmental Adaptation Biology, Ministry of Education, School of Life Sciences, Shandong University, Qingdao, Shandong 266237, China

Phosphorus deficiency severely limits crop production worldwide. While chemical fertilizers offer a temporary solution, their extensive use raises environmental concerns. Plants have evolved sophisticated strategies to cope with phosphorus limitation, ranging from enhanced expression of phosphate transporters to dramatic modifications of root architecture. Among these adaptations, cluster roots represent one of the most remarkable examples of root specialization. These bottle-brush–like structures, consisting of densely packed lateral rootlets (Figure), enhance phosphorus acquisition through increased surface area and concentrated exudation of organic acids and phosphatases that mobilize soil phosphorus.

**Figure.**
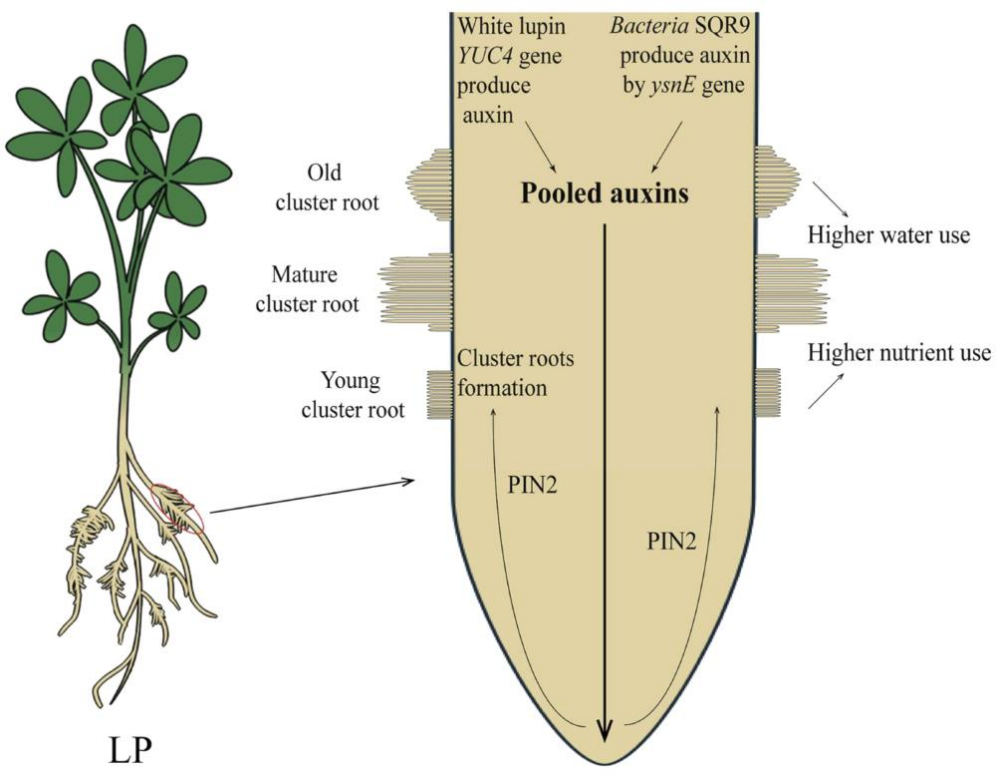
Integration of bacterial and plant auxin signals in cluster root formation under phosphorus deficiency. Under low-phosphorus conditions (LP), white lupin forms specialized cluster roots that enhance phosphorus acquisition. These cluster roots develop through 3 stages (young, mature, and old) and increase both nutrient and water uptake efficiency. The formation of cluster roots involves a sophisticated integration of auxin signals from both the plant and beneficial bacteria. Plant-derived auxin (produced by the YUC4 gene) and bacterial-derived auxin (produced by the ysnE gene in SQR9) form a pooled auxin reservoir. This pooled auxin is transported by the PIN2 auxin efflux carrier to promote cluster root formation. Figure was adapted from Yang et al. (2024).

White lupin (*Lupinus albus*) has emerged as a premier model for studying cluster root development, as it reliably forms these structures under phosphorus deficiency (O’Rourke et al. 2013). Although the mechanisms controlling cluster root formation remain largely unexplored, plants in natural settings interact with diverse soil microorganisms, raising the intriguing possibility that beneficial microbes might influence this crucial adaptive response. In this issue of *Plant Physiology*, Yang et al. unveil an interesting mechanism by which a plant growth-promoting bacterium enhances cluster root formation through coordinated auxin biosynthesis and transport pathways.

The authors first established that *Bacillus amyloliquefaciens* SQR9 could successfully colonize white lupin roots using GFP-labeled SQR9 strains. Under phosphorus-deficient conditions, SQR9 inoculation dramatically increased cluster root formation compared with non-inoculated plants. The enhancement was remarkably specific: cluster roots did not form under normal phosphorus supply regardless of bacterial presence, and the bacteria affected cluster root development without altering total root length. These observations suggested that SQR9 specifically augments the plant's adaptive response to phosphorus stress rather than inducing general root growth changes.

Through a series of genetic and pharmacological experiments, the authors demonstrated that bacterial auxin production via the *ysnE* gene pathway drives this enhancement, although its precise biochemical role in auxin biosynthesis remains to be determined. The application of exogenous indole-3-acetic acid increased cluster root number, while the auxin transport inhibitor 1-naphthylphthalamic acid significantly decreased it. By measuring auxin content in both bacterial cultures and plant tissues, they found that SQR9 produced significant amounts of indole-3-acetic acid in vitro, while plants inoculated with SQR9 showed elevated auxin levels in their roots. A bacterial mutant lacking *ysnE* produced 73.7% less auxin and failed to promote cluster root development. Using the auxin-responsive DR5rev::GFP reporter, they observed an increase in auxin responses in SQR9-inoculated roots. These findings were further supported by experiments with auxin transport inhibitor 1-naphthylphthalamic acid and biosynthesis inhibitor L-kynurenine, which reduced cluster root formation in a dose-dependent manner independent of SQR9 inoculation.

reverse transcription quantitative PCR analysis revealed that SQR9 inoculation significantly upregulated both *LaYUC4*, encoding a key auxin biosynthetic enzyme, and *LaPIN2*, encoding an auxin efflux transporter. Using virus-induced gene silencing, the authors demonstrated distinct roles for these genes: while *LaYUC4*-silenced plants retained partial responsiveness to SQR9, *LaPIN2* silencing completely abolished the bacterial enhancement of cluster root formation. These findings support a model where bacterial and plant-derived auxins form a “pooled” hormone reservoir that requires LaPIN2-mediated transport to promote cluster root development (Figure).

The specificity of the bacterial response to phosphorus status points to sophisticated regulatory mechanisms integrating nutrient sensing, microbial signals, and developmental responses. The authors found that sucrose levels in plant cells, previously implicated in cluster root formation, were unchanged by bacterial inoculation, suggesting that SQR9 acts through distinct pathways.

The evolutionary history of auxin biosynthesis reveals an interesting pattern—phylogenetic evidence suggests the pathway was acquired by ancestral land plants through horizontal gene transfer from bacteria (Yue et al. 2014). This gene transfer event may represent an ancient adaptation allowing plants to interact with and respond to soil microbes during the colonization of land. The coordinated action of bacterial and plant auxin systems in cluster root formation thus exemplifies how plants utilize and integrate microbial signals into their developmental programs, a capability that may extend to other plant species adapting to nutrient stress.

From a methodological perspective, this work provides a valuable template for studying plant–microbe interactions. The authors’ systematic combination of bacterial genetics, plant molecular biology, and detailed phenotypic analysis effectively dissected a complex biological process. Their successful application of VIGS in white lupin demonstrates the power of this technique for studying gene function in nonmodel species. This experimental framework could be particularly valuable for investigating other beneficial plant–microbe interactions where genetic tools are limited.

Several important questions emerge from this study. How do phosphorus-deficient conditions enable bacterial enhancement of cluster root formation? What upstream signals regulate the integration of bacterial and plant auxins? While this study focused on a single bacterial strain, natural soil communities contain diverse microorganisms that likely influence root development through multiple overlapping pathways. Understanding how plants integrate signals from multiple microbial partners could provide crucial insights into rhizosphere ecology and plant adaptation.

## Data Availability

No new data were generated or analysed in support of this research.
